# Tracking Active Fibroblast-Mediated Tissue Remodelling in Cardiomyopathies: Translational Insights from FAPI PET Imaging

**DOI:** 10.3390/ijms27198901

**Published:** 2026-10-07

**Authors:** Inês Fortuna, Janete Santos, Paula Soeiro, Raquel Machado, Ana Oliveira, Jorge Pereira, Giuseppe Limongelli, Teresa Faria, Elisabete Martins

**Affiliations:** 1 RISE-Health, Faculdade de Medicina, Universidade do Porto, 4200-319 Porto, Portugal; ifasilva@med.up.pt (I.F.); paula.soeiro@ulssjoao.min-saude.pt (P.S.);; 2 Faculty of Medicine, University of Porto, 4200-319 Porto, Portugal; 3 Nuclear Medicine, Centro Hospitalar Universitário de São João, E.P.E., 4200-319 Porto, Portugal; 4 Escola Superior de Saúde, Instituto Politécnico do Porto, 4200-072 Porto, Portugal; 5 Dipartimento di Scienze Mediche Traslazionali, Università della Campania “Luigi Vanvitelli”, AORN Colli, Ospedale Monaldi, 80131 Naples, Italy; 6 Department of Cardiology, Centro Hospitalar Universitário de São João, E.P.E., 4200-319 Porto, Portugal

**Keywords:** fibroblast activation protein, FAP inhibitor, FAPI PET, myocardial, myocardial fibrosis, cardiac remodelling, hypertrophic cardiomyopathy, cardiac magnetic resonance, PET/CMR, molecular imaging

## Abstract

Myocardial fibrosis is a major pathological component of cardiac remodelling and contributes to ventricular stiffness, impaired myocardial function, heart failure progression, and arrhythmic vulnerability across a broad spectrum of cardiomyopathies. In hypertrophic cardiomyopathy (HCM), fibrotic remodelling encompasses both diffuse interstitial matrix expansion and focal replacement fibrosis and is closely associated with disease severity and adverse clinical outcomes. Cardiac magnetic resonance (CMR), using late gadolinium enhancement (LGE) and quantitative parametric mapping, is central to non-invasive myocardial tissue characterization. LGE primarily identifies focal areas of replacement fibrosis and scar, whereas native T1, T2, and extracellular volume (ECV) mapping provide complementary information on diffuse myocardial and interstitial tissue abnormalities, including extracellular matrix expansion. However, these techniques characterize the structural and tissue-level consequences of myocardial injury and remodelling rather than directly visualizing the cellular processes underlying fibroblast activation. Fibroblast activation protein inhibitor positron emission tomography (FAPI PET) has emerged as a promising molecular imaging approach for assessing active fibroblast-mediated tissue remodelling. By targeting fibroblast activation protein (FAP), a cell-surface serine protease upregulated in activated fibroblasts and myofibroblasts. FAPI radiotracers may provide a molecular readout of accessible FAP expression that complements structural and tissue characterization by CMR. This review examines the cellular and molecular mechanisms underlying active myocardial remodelling, with particular emphasis on transforming growth factor-β1 (TGF-β1)/Smad signalling, fibroblast-to-myofibroblast transition, FAP expression, and extracellular matrix turnover. We critically evaluate the current clinical evidence for FAPI PET/CT and hybrid PET/CMR across cardiomyopathies, focusing on HCM while also considering ischemic, inflammatory, infiltrative, and other non-ischemic forms of cardiac remodelling. Particular attention is given to the relationship between myocardial FAPI uptake, CMR tissue characterization, ventricular function, circulating biomarkers, and calculated arrhythmic risk. Although initial studies support the complementary value of FAPI PET and CMR for characterizing active and established myocardial remodelling, the available evidence remains limited by small, predominantly single-centre cohorts, heterogeneous radiotracers and acquisition protocols, and the absence of validated quantitative thresholds or prospective outcome data. Larger longitudinal studies are required to determine whether FAPI PET can provide incremental prognostic information or serve as a reproducible imaging biomarker for disease progression and therapeutic response.

## 1. Introduction

Hypertrophic cardiomyopathy (HCM) is a common inherited heart condition affecting 1 in 200 to 500 people. It is primarily characterized by unexplained thickening of the left ventricular (LV) wall. While usually caused by an autosomal dominant genetic trait, HCM is etiologically diverse and can also stem from non-sarcomeric, metabolic, or mitochondrial disorders. Consequently, modern diagnosis requires combining structural imaging with systematic testing to rule out other causes of hypertrophy. Per current guidelines, an adult diagnosis requires a left ventricular wall thickness of ≥15 mm in at least one segment, though lesser thickening may qualify if supported by family history, genetics, or electrocardiogram (ECG) abnormalities [1,2,3].

At the histopathological level, HCM is characterized by cardiomyocyte hypertrophy, myocyte and myofibrillar disarray, abnormalities of the intramural coronary circulation, and heterogeneous extracellular matrix remodelling. Myocardial fibrosis may occur as diffuse interstitial or perimyocyte matrix expansion, perivascular fibrosis, focal replacement fibrosis, or mixed patterns. The relative contribution of these patterns varies according to disease stage and clinical phenotype. Fibroblast activation and extracellular matrix turnover are dynamic processes that may begin before overt LV systolic dysfunction. However, their reversibility depends on the biological composition and maturity of the fibrotic tissue. Early reactive interstitial remodelling may remain modifiable, whereas mature replacement fibrosis associated with irreversible cardiomyocyte loss generally represents persistent structural injury [4,5,6].

Activated fibroblasts and myofibroblasts are major cellular effectors of extracellular matrix synthesis, organization, degradation, and cross-linking in the injured myocardium. Their activity is regulated by the interaction of mechanical, inflammatory, neurohumoral, metabolic, and ischemic signals rather than by a single linear pathway. Persistent or dysregulated fibroblast activation promotes excessive deposition and maturation of extracellular matrix proteins, contributing to increased myocardial stiffness, impaired relaxation, and adverse ventricular restructuring [6].

Fibrosis drives several key features of HCM. Increased, cross-linked collagen stiffens the myocardium, impairing diastolic relaxation, while irregular interstitial and replacement scarring disrupts electrical signalling to create an arrhythmogenic substrate. Concurrently, coronary microvascular dysfunction—caused by arteriolar remodelling, lower capillary density, vessel compression, and high oxygen demand—limits blood flow. This relationship is complex and bidirectional: recurrent microvascular ischemia triggers cardiomyocyte injury and subsequent replacement fibrosis, while worsening hypertrophy, high filling pressures, and matrix scarring further impair myocardial perfusion [7,8].

Myocardial fibrosis is also a shared pathological response across ischemic, inflammatory, infiltrative, pressure-overload, and non-ischemic cardiomyopathies. However, the initiating mechanisms, cellular composition, temporal evolution, and functional consequences of restructuration differ according to the underlying disease. This heterogeneity is particularly relevant to molecular imaging because a signal associated with FAP-associated fibroblast activation may represent ischemic repair, inflammation-associated tissue remodelling, pressure-related adaptation, post-treatment injury, or another reparative process rather than a disease-specific form of fibrosis. Fibroblast activation protein (FAP)-targeted imaging should therefore be interpreted as a marker of an activated tissue-remodelling environment, rather than as a specific marker of HCM or myocardial fibrosis in isolation [9,10].

Cardiovascular magnetic resonance (CMR) is central to the non-invasive evaluation of HCM because it provides an accurate assessment of ventricular morphology, mass, volumes, systolic function, and myocardial tissue characteristics. Late gadolinium enhancement (LGE) is particularly well suited to detecting focal replacement fibrosis, whereas native T1 mapping and extracellular volume (ECV) quantification provide complementary quantitative information on diffuse myocardial abnormalities and interstitial matrix expansion. CMR should therefore not be regarded as a purely anatomical technique or as a modality that detects only end-stage scar. Its principal biological limitation in the present context is that it does not directly identify the cellular source of matrix production or distinguish ongoing active myocardial remodelling from established extracellular matrix expansion [2,4,11].

The extent of LGE holds proven prognostic value in HCM, especially for assessing ventricular arrhythmia risk. While an LGE burden of 15% of LV mass is commonly used to define extensive scarring and guide clinical decisions, this threshold is not a strict biological boundary. These estimates vary based on imaging techniques and post-processing methods, and significant clinical risk can still exist at lower burdens. Consequently, LGE should be interpreted as part of a comprehensive, multiparametric assessment rather than an isolated predictor of SCD or heart failure progression [2,12].

In clinically stable adult patients with HCM, contemporary guidelines indicate that repeat contrast-enhanced CMR every three to five years may be considered for periodic reassessment of SCD risk, or earlier when there is a significant change in clinical status. This interval reflects a clinical surveillance strategy and the expected evolution of disease; it should not be interpreted as evidence that CMR is intrinsically unable to detect earlier or more rapidly evolving myocardial abnormalities [2].

Against this background, FAP-targeted positron emission tomography (PET) has emerged as a molecular imaging strategy for investigating active tissue remodelling. FAP is expressed by subsets of activated fibroblasts and myofibroblasts in injured tissues, while expression is generally low in most quiescent adult myocardium. Radiolabeled FAP inhibitor (FAPI) permits in vivo visualization of tissues containing accessible FAP-expressing cells. Myocardial FAPI uptake should therefore be interpreted as a tracer-dependent surrogate of FAP-associated cellular activity [13,14,15,16].

Initial clinical studies in HCM have demonstrated intense and spatially heterogeneous myocardial FAPI uptake, including uptake in regions with established CMR abnormalities and in some segments without detectable LGE or overt hypertrophy. Quantitative FAPI measures have shown associations with N-terminal pro-B-type natriuretic peptide (NT-proBNP), high-sensitivity cardiac troponin I, LV function and myocardial deformation. Integrated PET/CMR studies have identified overlapping and discordant molecular and structural imaging phenotypes, supporting the hypothesis that FAPI PET and CMR interrogate related but non-identical components of myocardial remodelling. Wang et al. assessed 50 patients with symptomatic non-ischemic cardiomyopathies and showed significance correlation of FAPI uptake with enlarged atrial diameter and LV ejection fraction [17].

These observations do not establish that FAPI PET consistently detects fibrogenesis before the development of parametric CMR abnormalities or predicts adjudicated clinical events. The current evidence remains predominantly based on small observational cohorts and heterogeneous radiotracers. Furthermore, FAP expression is not specific to cardiac fibrosis and may occur in inflammatory, reparative, degenerative, post-treatment, and neoplastic tissue environments. FAPI PET should therefore currently be regarded as an investigational molecular imaging approach with potential to complement, rather than replace, established structural, functional, and tissue-characterization techniques [18,19].

### Objectives

This review summarizes current imaging strategies for the assessment of myocardial fibrosis, with an emphasis on the molecular pathways of active myocardial remodelling and the emerging clinical role of FAPI PET/CT.

The review further appraises the available clinical evidence linking myocardial FAPI uptake with structural tissue abnormalities, ventricular function, myocardial deformation, circulating biomarkers, and calculated arrhythmic risk. Finally, we discuss the principal methodological and translational limitations of FAPI imaging—including target specificity, tracer heterogeneity, image acquisition and quantification, reproducibility, radiation exposure, and the absence of validated prognostic thresholds—and consider the evidence required to establish its future role in disease phenotyping, longitudinal monitoring, and the assessment of antifibrotic therapeutic response.

## 2. Molecular and Cellular Pathways of Active Myocardial Remodelling

Myocardial fibrosis is a complex response to cardiac injury and chronic biomechanical stress. Resident cardiac fibroblasts are central effectors of extracellular matrix (ECM) homeostasis and remodelling. In response to mechanical, neurohumoral, inflammatory, metabolic, or ischemic stimuli, these cells may proliferate and acquire a spectrum of activated phenotypes, including highly secretory and contractile myofibroblast states. Fibroblast activation is therefore more accurately understood as a heterogeneous phenotypic transition than as the uniform conversion of all quiescent fibroblasts into a single myofibroblast population. Cardiomyocytes, endothelial cells, pericytes, macrophages, lymphocytes, and mast cells also modulate the fibrotic response through paracrine signalling, protease release, and regulation of fibroblast function [6,20].

Transforming growth factor-β (TGF-β) is a central regulator of cardiac fibroblast activation, although it is not the sole molecular driver of myocardial fibrosis. Binding of TGF-β isoforms to their receptors promotes phosphorylation of Smad2 and Smad3, formation of transcriptional regulatory complexes, and expression of genes involved in fibroblast activation, collagen synthesis, and ECM organization. Experimental lineage-tracing and fibroblast-specific genetic studies have identified TGF-β/Smad2/3 signalling in tissue-resident cardiac fibroblasts as a major mediator of the fibrotic response. Canonical Smad signalling nevertheless interacts with non-canonical pathways, mechanotransduction, inflammatory mediators, and neurohumoral systems, and its biological effects depend on the nature, intensity, duration, and temporal stage of myocardial injury. Khalil et al. specifically demonstrated that fibroblast-restricted disruption of TGF-β receptor or Smad2/3 signalling markedly attenuated the fibrotic gene programme in experimental cardiac injury [21].

Activated fibroblasts and myofibroblasts synthesize fibrillar collagens, predominantly collagen types I and III, together with fibronectin, matricellular proteins, proteoglycans, glycosaminoglycans, and other non-fibrillar ECM components. They also regulate matrix degradation, organization, and cross-linking through matrix metalloproteinases, tissue inhibitors of metalloproteinases, other proteases, and collagen cross-linking enzymes. These processes influence not only myocardial stiffness but also cell–matrix signalling, growth-factor availability, electrical propagation, microvascular function, and cardiomyocyte survival. Fibroblast activation may initially have an adaptive or reparative role by preserving tissue integrity; however, persistent or dysregulated activation promotes excessive matrix accumulation, increased collagen cross-linking, impaired ventricular compliance, and adverse remodelling [6,10].

In HCM, fibrotic remodelling results from interactions between primary sarcomeric abnormalities, altered myocardial energetics, abnormal mechanotransduction, cardiomyocyte stress and death, microvascular dysfunction, ischemia, and neurohumoral and inflammatory signalling. The resulting fibrotic phenotype is spatially heterogeneous and may include interstitial or perimyocyte fibrosis, perivascular fibrosis, focal replacement fibrosis, and mixed patterns. These forms frequently coexist and vary according to disease stage and clinical phenotype rather than representing mutually exclusive categories. Microvascular ischemia is an important contributor to cardiomyocyte injury and subsequent replacement fibrosis, whereas diffuse interstitial remodelling may develop around viable cardiomyocytes and precede overt LV systolic dysfunction [4,5,8].

Histopathological evidence indicates that the relative contribution of these fibrotic patterns changes across the HCM disease spectrum. Interstitial and perimyocyte fibrosis are prominent in septal myectomy specimens from patients with obstructive HCM and preserved or hyperdynamic LV systolic function [4,5].

From an imaging perspective, LGE predominantly identifies focal regions of expanded extracellular space associated with replacement fibrosis. It does not directly identify the cellular mechanisms responsible for matrix deposition and may underestimate diffuse microscopic, interstitial, or perimyocyte fibrosis. Native T1 and ECV mapping provide complementary quantitative information on diffuse myocardial abnormalities and interstitial expansion, but neither directly measures FAP-associated fibroblast activation or active collagen synthesis. Cellular activation, matrix production, interstitial expansion, collagen maturation, and replacement scar should therefore be understood as partially overlapping biological processes rather than as strictly sequential and mutually exclusive stages [4,6].

The potential reversibility of myocardial fibrosis depends on its biological composition, cross-linking, spatial organization, and stage. Early reactive interstitial remodelling may remain modifiable when the underlying stimulus is removed or effectively treated. However, reversibility cannot be assumed when collagen deposition is extensive, highly cross-linked, or accompanied by irreversible cardiomyocyte loss. Mature replacement fibrosis generally represents a persistent structural scar, although fibroblast activity and matrix turnover may continue within the scar border zone or in the surrounding chronically injured myocardium. Reactive interstitial fibrosis and replacement fibrosis should consequently not be regarded as completely reversible and entirely biologically inert states, respectively [6,10].

FAP is a membrane-associated serine protease. FAP exhibits post-proline dipeptidyl peptidase and endopeptidase activity and may contribute to fibroblast migration, gelatin degradation, wound healing, and ECM remodelling. Experimental studies after myocardial infarction demonstrated FAP expression in activated cardiac fibroblasts and showed that TGF-β1 can induce its expression. However, FAP expression is neither universal among all activated fibroblasts nor specific to cardiac fibrosis, and its functional contribution to matrix synthesis, degradation, migration, and tissue repair remains incompletely understood [13,14,22].

The signal obtained by PET is a result of cellular activity FAPI associated, which does not reflect the number of fibroblasts, collagen synthesis, ECM volume, or histological fibrosis. Therefore, the measurement given by the FAPI tracer to be an indirect measurement [14,15,16,23].

Initial clinical studies in HCM have demonstrated intense and spatially heterogeneous myocardial FAPI uptake, including activity in hypertrophied and non-hypertrophied regions, in myocardial segments with CMR abnormalities, and in some segments without detectable LGE. Wang et al. reported heterogeneous myocardial [^18^F]F-FAPI activity extending beyond hypertrophied segments and an association with calculated five-year HCM Risk-SCD. Zhang et al. showed that FAPI-positive segments not identified as abnormal by conventional CMR tissue-characterization criteria could nevertheless exhibit impaired myocardial strain. Integrated [^68^Ga]Ga-FAPI-04 PET/CMR further demonstrated overlapping and discordant FAPI, LGE, native T1, T2, ECV, and strain abnormalities [17,18,23].

**Figure 1:**
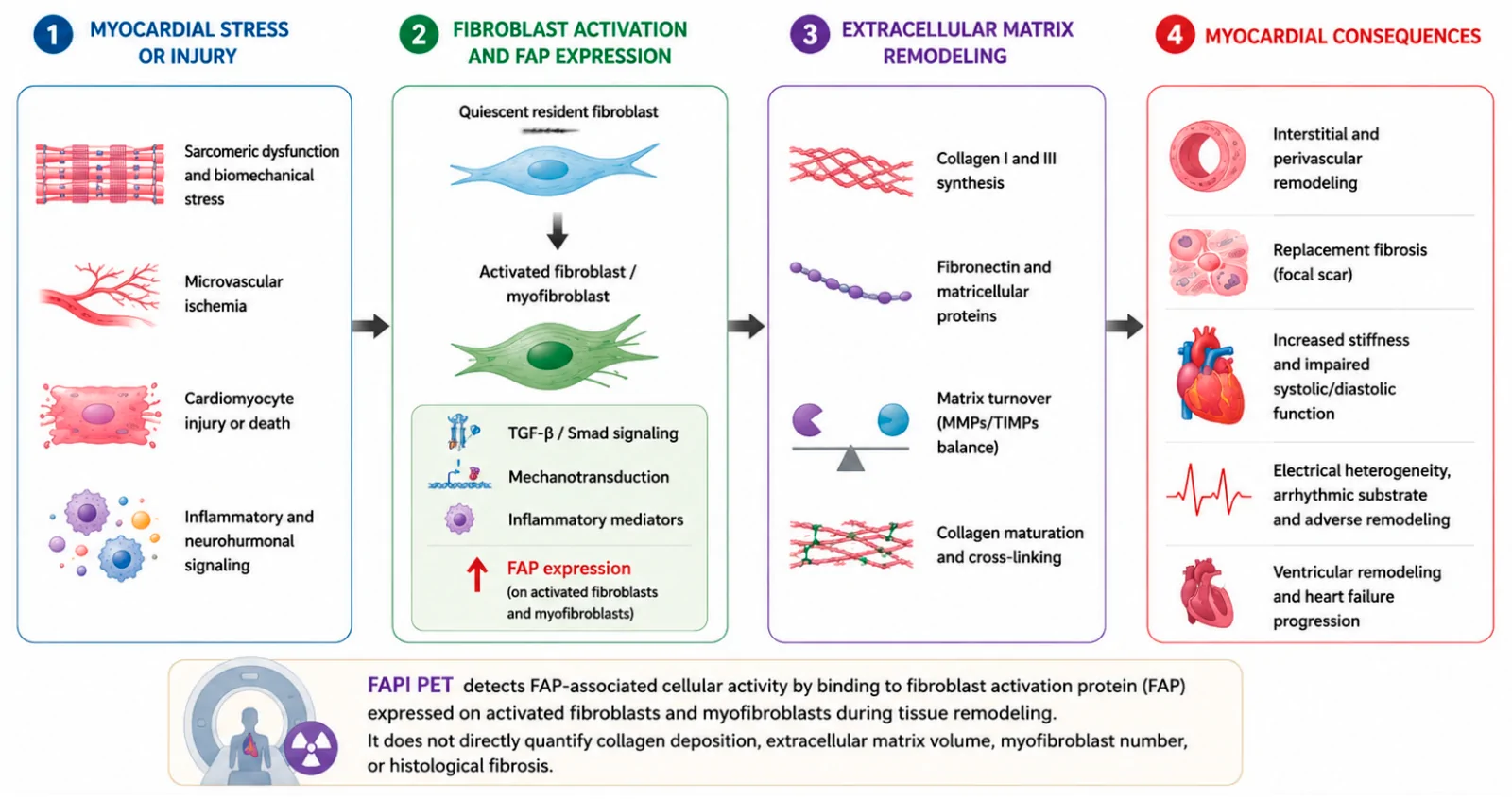
Molecular and cellular pathways linking myocardial injury to FAP-associated fibroblast activation and extracellular matrix remodelling. Sarcomeric dysfunction, biomechanical stress, microvascular ischemia, cardiomyocyte injury, and inflammatory or neurohumoral signalling promote activation of resident cardiac fibroblasts through interacting pathways that include TGF-β/Smad signalling, mechanotransduction, and inflammatory mediators. Activated fibroblasts and myofibroblasts may upregulate FAP and contribute to extracellular matrix synthesis, turnover, maturation, and cross-linking. Persistent or dysregulated remodelling may result in interstitial and perivascular matrix expansion, replacement fibrosis, increased myocardial stiffness, impaired ventricular function, electrical heterogeneity, and adverse cardiac remodelling. FAPI PET provides an indirect, tracer-dependent signal associated with accessible FAP expression in activated stromal cells; it does not directly quantify collagen deposition, extracellular matrix volume, myofibroblast number, or histological fibrosis. The pathways shown are interconnected and should not be interpreted as a strictly linear or universally sequential process. Abbreviations: ECM, extracellular matrix; FAP, fibroblast activation protein; FAPI, fibroblast activation protein inhibitor; MMPs, matrix metalloproteinases; PET, positron emission tomography; TGF-β, transforming growth factor-β; TIMPs, tissue inhibitors of metalloproteinases.

The molecular and cellular pathways linking myocardial stress or injury to fibroblast activation, FAP expression, extracellular matrix remodelling, and downstream myocardial consequences are summarized in Figure 1.

## 3. Strategies for Assessing Myocardial Remodelling

CMR is the reference non-invasive imaging modality for the assessment of cardiac morphology, ventricular function, and myocardial tissue characteristics. CMR should not be regarded as a purely anatomical technique. Cine imaging enables accurate quantification of ventricular volumes, mass, wall thickness, and systolic function, whereas LGE, native T1 mapping, T2 mapping, and ECV quantification provide distinct and complementary information on myocardial composition and injury. Contemporary guidelines place CMR at the centre of diagnostic evaluation [22], differential diagnosis, and risk assessment in HCM. CMR is particularly valuable because it integrates morphology, function, and tissue characterization within a single examination [1,2,11].

LGE is particularly well suited to detecting focal replacement fibrosis because gadolinium-based contrast agents distribute within the extracellular space and produce delayed enhancement in regions where that space is expanded. Its sensitivity, however, depends on the contrast between abnormal and relatively preserved reference myocardium and is influenced by acquisition, signal-thresholding, segmentation, and quantification methods. Native T1 mapping and ECV quantification complement LGE by providing quantitative information on diffuse myocardial abnormalities and interstitial matrix expansion, including in patients without visually detectable focal enhancement. CMR therefore characterizes substantially more than macroscopic or end-stage scar. Its principal biological limitation in the present context is that it does not directly identify the cellular source of ECM production or distinguish ongoing FAP-associated-fibroblast activation from established matrix expansion [4,11].

PET provides molecular information through the biodistribution, tissue uptake, and retention of radiolabeled probes. The biological interpretation of a PET signal depends on the molecular target, tracer affinity and pharmacokinetics, tissue perfusion, vascular permeability, clearance, uptake interval, image reconstruction, and analysis methodology. PET generally has lower spatial resolution than CMR, but its high molecular sensitivity permits the non-invasive investigation of biological processes that cannot be directly visualized using conventional structural or parametric imaging [24,25].

Integrated [^68^Ga]Ga-FAPI 04 PET/CMR has further demonstrated both overlapping and discordant molecular and structural abnormalities in HCM. Ding et al. studied 20 patients with HCM and 11 matched controls. Increased myocardial FAPI uptake was present in 19 of 20 patients and involved 156 of 320 myocardial segments, whereas LGE was present in 95 segments. Segments positive for both FAPI uptake and LGE showed greater wall thickness, higher native T1, T2, and ECV values, and more pronounced myocardial strain impairment than segments with isolated or absent abnormalities. These findings support biological complementarity between FAPI PET and CMR rather than a simple temporal model in which FAPI positivity universally precedes CMR abnormalities [18,26,27]. Similarly, Zhang et al., using [^18^F]]F-FAPI PET also in HCM patients and They obtained results showing that PET detects more myocardial tissue relative to CMR techniques. However, these findings do not establish that such segments necessarily represent early fibrosis or will subsequently develop LGE-positive scar [23].

**Figure 2:**
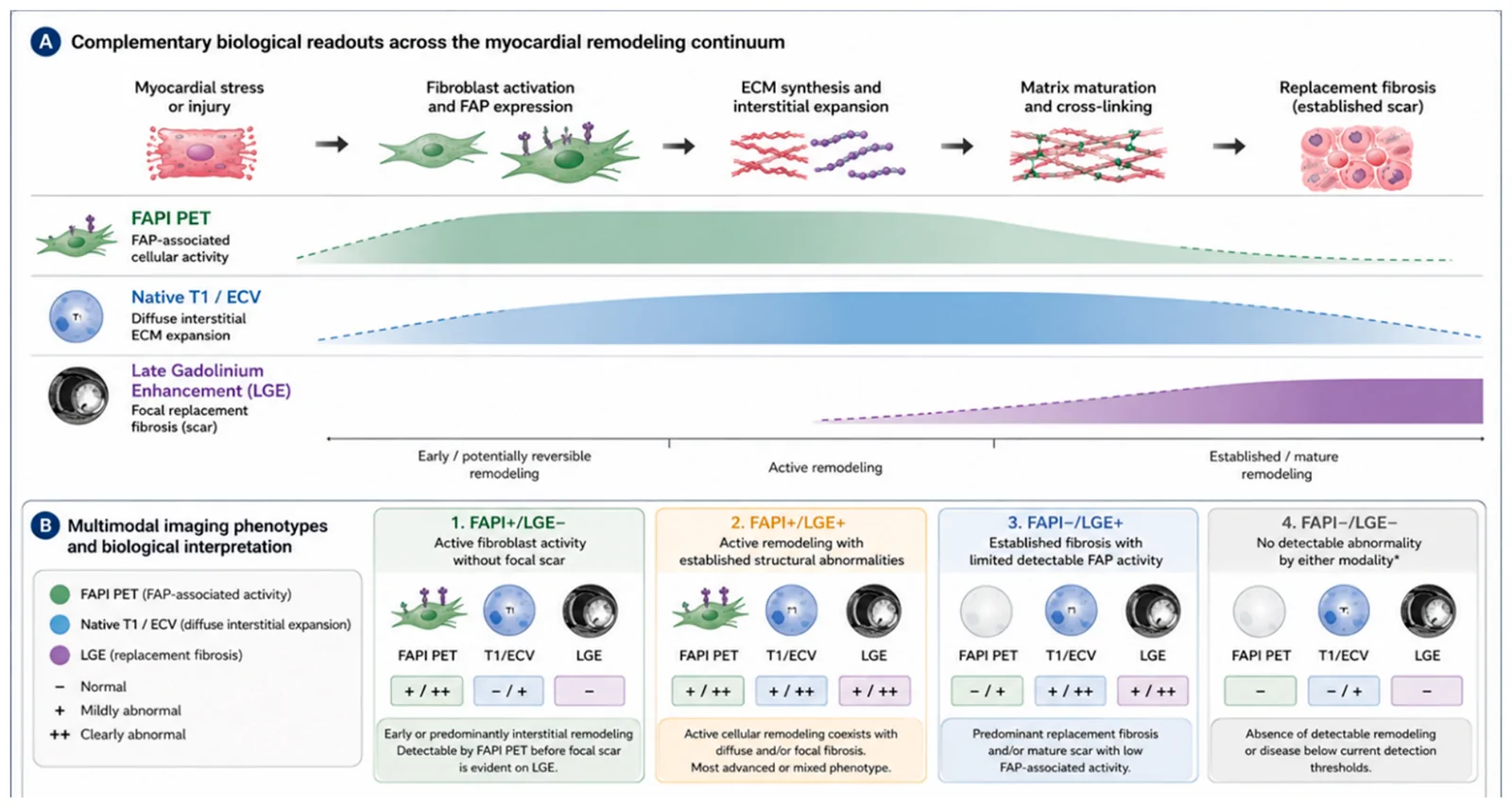
Complementary imaging of myocardial remodelling by FAPI PET and cardiovascular magnetic resonance. Complementary biological readouts of FAPI PET and cardiovascular magnetic resonance across myocardial remodelling. (**A**) Schematic representation of the myocardial remodelling continuum and the distinct, partially overlapping biological information provided by FAPI PET, native T1/ECV mapping, and LGE. FAPI PET reflects molecular signals associated with activated fibroblasts, whereas native T1 and ECV provide information related to myocardial tissue abnormalities and diffuse extracellular/interstitial expansion, and LGE primarily identifies areas of focal myocardial injury and replacement fibrosis. These modalities therefore provide complementary, rather than interchangeable, information and may differ in their spatial and temporal expression during myocardial remodelling. (**B**) Conceptual multimodal imaging phenotypes and their biological interpretation. Abbreviations: CMR, cardiovascular magnetic resonance; ECM, extracellular matrix; ECV, extracellular volume fraction; FAP, fibroblast activation protein; FAPI, fibroblast activation protein inhibitor; LGE, late gadolinium enhancement; PET, positron emission tomography. * A normal multimodality profile does not exclude early or subclinical disease.

Figure 2 illustrates the biological pathways driving myocardial fibrosis and outlines how these processes are detected and monitored via CMR and FAPI-PET. Table 1 compares the diagnostic capabilities of both modalities, highlighting their respective strengths in identifying distinct types of cardiac fibrosis.

## 4. FAPI PET—Identification of Active Myocardial Remodelling

The interpretation of myocardial FAPI uptake as a candidate imaging biomarker requires consideration of both biological and technical determinants. Quantitative approaches reported in cardiovascular studies include maximum and mean standardized uptake values (SUVmax and SUVmean), target-to-background ratios, the number or proportion of FAPI-positive myocardial segments, FAPI-positive myocardial volume, and composite indices integrating signal intensity and spatial extent. These measures are not interchangeable and may be influenced by tracer identity and affinity, administered activity, uptake interval, blood-pool clearance, tissue perfusion, image reconstruction, cardiac and respiratory motion, partial-volume effects, myocardial segmentation, and the choice of reference tissue. No standardized cardiac FAPI metric, normal reference range, positivity threshold, or prognostically validated cut-off has yet been established [17,18,28].

Preliminary clinical evidence indicates that myocardial FAPI uptake is associated with selected markers of HCM severity. These findings suggest that a greater myocardial FAPI burden accompanies increased hemodynamic stress, cardiomyocyte injury, and clinical disease severity. However, the study did not demonstrate that FAPI uptake directly measures myocardial injury or independently predicts SCD or ventricular arrhythmic events [17].

In an earlier prospective preliminary study, Zhang et al. evaluated 22 patients with HCM and 22 age- and sex-matched controls using [^18^F]F-FAPI PET/CT. Within the HCM group, the spatial extent of myocardial uptake showed moderate associations with NT-proBNP and creatine kinase–myocardial band (CK-MB). These relationships were reported for the extent of FAPI-positive myocardium rather than for SUV_max or target-to-background ratio alone, illustrating that different quantitative constructs may have different biological associations. The absence of comparable associations between the assessed biomarkers and CMR parameters in this small exploratory cohort does not establish that FAPI PET has superior sensitivity over CMR [29].

Integrated [^68^Ga]Ga-FAPI-04 PET/CMR has provided additional evidence linking FAPI uptake with structural and functional myocardial abnormalities. In the study by Ding et al., segments positive for both FAPI uptake and LGE showed greater wall thickness, higher native T1, T2, and ECV values, and more pronounced strain impairment than segments with isolated or absent abnormalities. Myocardial FAPI measures were also evaluated in relation to calculated SCD and atrial-fibrillation risk estimates. These findings support the complementary biological information provided by molecular and CMR-based tissue characterization, but they do not validate FAPI uptake as a circulating inflammatory biomarker, an independent predictor of clinical events, or a disease-specific measure of fibrosis [18].

NT-proBNP, high-sensitivity cardiac troponin, and CK-MB provide clinically relevant but biologically non-specific information. NT-proBNP predominantly reflects myocardial wall stress and hemodynamic load, whereas cardiac troponins and CK-MB indicate cardiomyocyte injury to differing degrees of analytical sensitivity and specificity. None directly measures active myocardial remodelling, FAP expression, collagen synthesis, or ECM turnover [30,31,32].

## 5. Clinical Evidence and Potential Applications of FAPI PET in Cardiovascular Disease

FAP-targeted molecular imaging has been investigated across a growing range of cardiovascular conditions, including acute myocardial infarction, hypertrophic and dilated cardiomyopathies, pressure-overload remodelling, pulmonary hypertension, myocarditis, cardiac sarcoidosis, myocarditis with immune checkpoint inhibitor (ICI) and cardiac amyloidosis. The maturity of the evidence varies substantially across disease settings [19,22,33,34,35]. Table 2 summarizes the main challenges (like Key biological, technical, clinical, and regulatory challenges in cardiovascular) facing FAPI-PET as a tool for identifying and assessing cardiac involvement in cardiovascular diseases in order to obtain the necessary evidence required for biomarker validation and clinical implementation.

### 5.1. Cardiovascular Conditions

FAPI imaging has also been evaluated in dilated cardiomyopathy and broader non-ischemic cardiomyopathy populations. A 2025 study comparing [^18^F]]F-FAPI PET with CMR and histopathology in dilated cardiomyopathy demonstrated increased myocardial uptake, more FAPI-positive than LGE-positive segments, and strong associations with histological markers of FAP—associated fibroblast activation and myocardial tissue abnormalities [36]. This provides more direct biological validation than imaging-only studies, although larger cohorts and outcome analyses remain necessary.

Studies in pulmonary arterial and chronic thromboembolic pulmonary hypertension have demonstrated FAPI activity in the RV and pulmonary arterial wall, with associations with pressure overload, RV dysfunction, and clinical severity. These observations support the investigation of FAPI imaging as a marker of pressure-overload remodelling but do not establish earlier diagnosis or superior prognostic performance compared with echocardiography or CMR [33].

Myocardial FAPI uptake has also been reported in cardiac sarcoidosis and has been demonstrated to increase [^68^Ga]Ga-FAPI-04 uptake associated with active myocardial remodelling. In cardiac sarcoidosis, Siebermair et al. reported persistent [^68^Ga]Ga-FAPI-46 uptake in previously inflamed and LGE-positive regions despite resolution of fluorodeoxyglucose (FDG) activity after immunosuppression. The observation influenced the treating team’s decision to continue therapy, but it remains a single case and cannot establish the appropriate duration or intensity of immunosuppressive treatment [37].

### 5.2. Myocardial Fibroblast Activation After Acute Myocardial Infarction

Acute myocardial infarction provides the clearest clinical model for investigating the temporal relationship between myocardial injury, fibroblast activation, ECM deposition, and scar formation. Experimental studies have demonstrated marked FAP expression in activated fibroblasts within infarct and peri-infarct tissue during the reparative phase after ischemic injury. These findings provide the biological basis for using FAPI imaging to investigate post-infarction remodelling, although signal intensity and distribution depend on infarct size, reperfusion status, tracer characteristics, and imaging time point [38].

In a single-centre study of 35 patients imaged within 11 days of acute myocardial infarction, Diekmann et al. found that the myocardial territory with increased [^68^Ga]Ga-FAPI-46 uptake was substantially larger than both the perfusion defect measured by single-photon emission computed tomography and the infarct region identified by LGE. The mean FAPI-positive volume represented approximately 58% of the LV, compared with 23% for the perfusion defect and approximately 28% for the LGE-defined infarct. These findings indicate that post-infarction FAP-associated activity may extend beyond the necrotic core into peri-infarct and apparently non-infarcted myocardium. Measures of spatial FAPI burden were also associated with subsequent ventricular dysfunction, although the study did not establish a validated prognostic threshold [39].

In a separate prospective study of 14 patients with reperfused ST-segment elevation myocardial infarction, Xie et al. compared early [^18^F]F-FAPI imaging with CMR and obtained follow-up CMR in 10 participants. FAPI activity was associated with several CMR measures of myocardial injury and remodelling, and exploratory analyses suggested that the spatial extent of uptake may be more informative than maximum signal intensity alone. However, the small sample and incomplete longitudinal imaging preclude firm conclusions regarding prognostic utility [40].

### 5.3. Myocardial Phenotyping and Risk Assessment in Hypertrophic Cardiomyopathy

HCM is currently the most extensively studied non-ischemic application of cardiac FAPI PET. Available studies consistently demonstrate increased and spatially heterogeneous myocardial uptake, frequently extending beyond regions of overt hypertrophy or LGE. These findings suggest that FAPI PET may identify a component of myocardial remodelling that is not fully represented by conventional morphological or structural tissue-characterization measures [23,41].

In the prospective study by Wang et al., 50 patients with HCM and 22 healthy controls underwent [^18^F]F-FAPI PET/CT. All patients with HCM showed increased and heterogeneous myocardial activity compared with controls. The number of FAPI-positive segments exceeded the number of hypertrophied segments, and uptake was also observed in non-hypertrophied myocardium. FAPI amount was associated with NT-proBNP, high-sensitivity cardiac troponin I, left atrial diameter, and LV systolic function and showed a modest positive correlation with the calculated five-year HCM Risk-SCD score. This was an association with a calculated risk model rather than with prospectively adjudicated sudden death or ventricular arrhythmic events [17,26,32,42].

Zhang et al. subsequently evaluated 49 patients with HCM using [^18^F]F-FAPI PET/CT and CMR feature-tracking analysis. Across 766 myocardial segments, the number of FAPI-positive segments exceeded both the number of hypertrophied segments and the number of segments with abnormal CMR tissue characterization. Among segments without conventional CMR abnormalities, FAPI-positive regions showed greater impairment of myocardial strain than FAPI-negative regions. These findings support the functional relevance of some PET-positive/CMR-negative segments but do not establish that all such segments represent early fibrosis or will inevitably progress to LGE-positive scar [23].

Cardiac PET/MRI integrated with [68Ga]Ga-FAPI-04 provided further evidence of overlapping and discordant imaging phenotypes. Ding et al. reported increased myocardial uptake in the vast majority of patients, identifying significantly more myocardial segments compared to segments positive for late gadolinium enhancement (LGE). Segments positive for both FAPI uptake and LGE demonstrated greater wall thickness, more abnormal mapping values, higher ECV, and greater impairment of myocardial deformation. These findings suggest that combined molecular and structural abnormalities may characterize more advanced myocardial involvement. Associations with SCD and atrial-fibrillation risk scores, nevertheless, concerned calculated estimates rather than observed clinical events [18].

Histopathological validation remains limited. Li et al. reported two patients with obstructive HCM who underwent [^18^F]F-FAPI PET/CT before septal myectomy. Both patients demonstrated heterogeneous septal uptake, and resected tissue showed activated fibroblasts and variable fibrosis. This case series supports the biological plausibility of myocardial FAPI uptake but cannot establish quantitative histological validity, diagnostic accuracy, or surgical utility [41].

More recently, a retrospective two-centre proof-of-concept study evaluated FAPI PET radiomics in 92 patients with HCM. The radiomics model captured heterogeneity of myocardial uptake and showed moderate internally validated discrimination for categories of calculated SCD risk. Performance decreased after bootstrap correction and became broadly comparable to a conventional FAPI metric. The endpoint was predicted risk rather than observed SCD, and external validation is required. This study supports FAPI PET as a research tool for molecular phenotyping, including SCD risk stratification in HCM [43].

To synthesize the recent studies in which FAPI PET was used to assess cardiac involvement in patients with HCM, we identified, collected, and analyzed the available clinical evidence. Table 3 summarizes selected clinical studies evaluating FAPI-targeted imaging in HCM, with particular emphasis on the assessment of active fibroblast-mediated tissue and myocardial remodelling. For each study, the table presents the study design and population, the tracer and imaging platform used, the principal findings, and the major limitations. To this end, a structured literature search was conducted in the PubMed database using the following search strategy:

(((Hypertrophic cardiomyopathy) OR (“Hypertrophic cardiomyopathy” [MeSH Terms]) OR (“cardiomyopathy, hypertrophic” [MeSH Terms]) OR (hypertrophic cardiomyopathy)) AND (FAPI)).

Studies conducted in animal models or in vitro were excluded, as were narrative reviews, systematic reviews, and meta-analyses, in order to ensure the inclusion of original clinical investigations only. All studies included were from January of 2020 until March of 2026.

### 5.4. Longitudinal Assessment and Therapeutic-Response Monitoring

The capacity to visualize an active cellular component of myocardial remodelling makes FAPI imaging conceptually attractive for longitudinal assessment. Serial post-infarction studies demonstrate that myocardial FAPI activity changes over time and may persist beyond the acute healing phase. Delayed PET/MR studies have also shown persistent uptake after myocardial infarction, supporting the presence of ongoing activation of myocardial remodelling in selected patients. A similar biological rationale may apply to immune checkpoint inhibitor (ICI)-associated myocarditis, in which T-cell-mediated cardiomyocyte injury can trigger activation of the cardiac stroma, including fibroblast-to-myofibroblast differentiation and increased FAP expression. FAPI PET may therefore provide a means of detecting the fibroblast activation associated with this inflammatory myocardial injury, although the clinical and prognostic significance of this signal remains to be established [22,32,34].

However, temporal variation in uptake is not equivalent to validation as a treatment-response biomarker. A reduction or increase in signal may reflect the natural evolution of tissue repair, changes in perfusion or tracer delivery, altered fibroblast phenotype, differences in acquisition or reconstruction, or a genuine pharmacological effect [34,37,44].

The two-patient HCM histopathological series did not assess therapeutic response, and the cardiac sarcoidosis report represented an individual clinical decision rather than longitudinal biomarker validation. These publications do not support individualized dose optimization or routine treatment guidance [25,37].

FAPI PET may nevertheless have future value as a pharmacodynamic or treatment-response imaging biomarker, provided that prospective studies demonstrate analytical reproducibility, temporal stability, biological responsiveness, and an association between treatment-induced changes in uptake and clinically meaningful outcomes [34].

**Figure 3:**
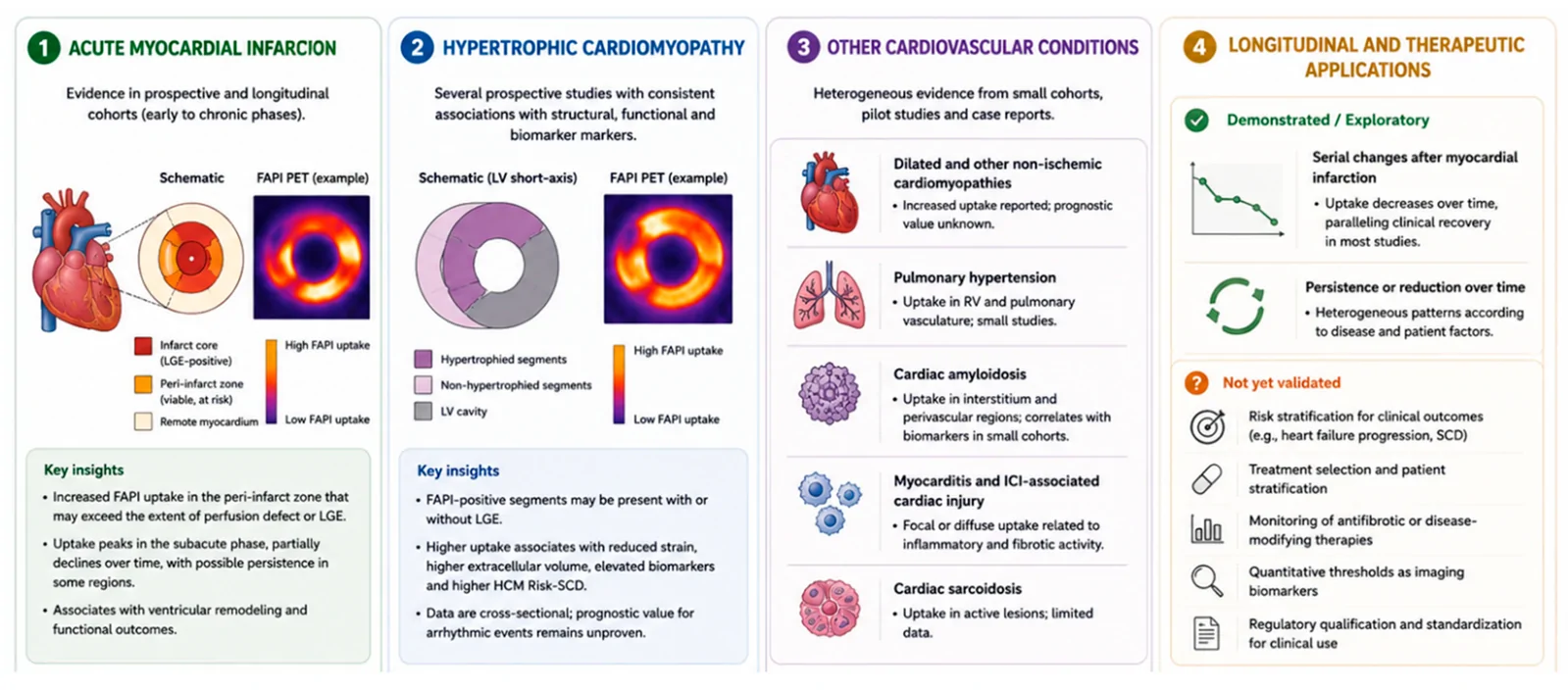
Current cardiovascular applications and evidence maturity of FAPI-targeted PET imaging. Overview of the main disease contexts in which FAPI PET has been investigated, the typical imaging patterns, key insights, and the current level of evidence. While several studies support the ability of FAPI PET to detect FAP-associated cellular activity during myocardial remodelling, its incremental prognostic value and role in guiding therapy remain unproven and require prospective validation. Abbreviations: CMR, cardiovascular magnetic resonance; FAPI, fibroblast activation protein inhibitor; FAP, fibroblast activation protein; HCM, hypertrophic cardiomyopathy; ICI, immune checkpoint inhibitor; LGE, late gadolinium enhancement; LV, left ventricle; PET, positron emission tomography; RV, right ventricle; SCD, sudden cardiac death.

The principal cardiovascular applications of FAPI PET and the current maturity of the supporting evidence are summarized in Figure 3.

## 6. Future Perspectives, Challenges, and Research Priorities

FAP-targeted PET imaging provides a conceptually attractive approach for investigating active myocardial remodelling. Current studies suggest that myocardial FAPI uptake may identify spatially heterogeneous FAP-associated cellular activity that extends beyond regions of hypertrophy or LGE in some patients with HCM. Integrated PET/CMR studies further demonstrate that increased molecular tracer uptake, parametric mapping abnormalities, impaired myocardial deformation, and replacement fibrosis may coexist in different combinations. These observations support the complementary biological information provided by FAPI PET and CMR but derive predominantly from small, exploratory cohorts and do not establish routine diagnostic or prognostic utility [17,18,23,45].

The future clinical value of FAPI PET will depend on its ability to address relevant biological or clinical questions not adequately resolved by established imaging methods. Potential applications include characterization of active myocardial remodelling, refinement of disease phenotypes, assessment of the spatial relationship between cellular activity and structural tissue abnormalities, and longitudinal evaluation of disease progression or treatment response. The presence of FAPI uptake outside LGE-positive regions is biologically relevant but does not establish that these regions exclusively represent early, reversible, or progressive fibrosis. Longitudinal studies are required to define the natural history of different multimodal phenotypes, including FAPI-positive/LGE-negative, FAPI-positive/LGE-positive, and FAPI-negative/LGE-positive myocardium.

Hybrid PET/CMR is particularly suited to mechanistic investigation because it permits near-simultaneous assessment of FAP-associated tracer uptake, ventricular morphology and function, myocardial deformation, edema-sensitive parameters, interstitial expansion, and replacement fibrosis. Near-simultaneous acquisition reduces temporal discrepancies between modalities and supports spatial co-registration. However, it does not eliminate cardiac or respiratory motion, partial-volume effects, blood-pool activity, segmentation error, or PET/MR attenuation-correction limitations [44].

In HCM, segments with abnormalities on both FAPI PET and CMR have shown greater wall thickness, higher native T1 and T2 values, greater ECV expansion, and more impaired strain than segments with isolated or absent abnormalities. These findings suggest that concordant molecular and structural abnormalities may identify more advanced myocardial involvement. Nevertheless, no study has yet demonstrated that integrated FAPI PET/CMR improves diagnostic accuracy, prognostic discrimination, reclassification, or clinical decision-making compared with optimized CMR alone.

FAPI imaging may also have potential as a pharmacodynamic biomarker in trials of therapies intended to modify myocardial remodelling [9]. In principle, serial quantification could determine whether an intervention is associated with a reduction, persistence, or redistribution of FAP-associated activity before measurable structural changes become apparent. However, temporal changes in uptake cannot automatically be interpreted as treatment effects. They may reflect the natural evolution of tissue repair, changes in perfusion or tracer delivery, altered FAP expression or fibroblast phenotype, differences in uptake interval or reconstruction, or measurement variability.

Current evidence does not support the use of FAPI PET for selecting antifibrotic therapy, optimizing individual drug doses, or serving as a validated surrogate endpoint. Siebermair et al. reported a single patient with cardiac sarcoidosis in whom persistent [^68^Ga]Ga-FAPI-46 uptake was observed after immunomodulatory treatment despite resolution of FDG activity. This finding suggested persistent fibroblast-associated tissue activity but did not validate therapeutic monitoring, treatment duration, or dose adjustment [37,45].

For FAPI PET to become a credible treatment-response biomarker, future studies must demonstrate analytical reproducibility, establish expected biological variation in untreated disease, define minimum detectable and clinically meaningful changes, and show that treatment-induced changes in uptake correspond to independent biological effects and subsequent clinical outcomes. Qualification as a surrogate endpoint would require stronger evidence that changes in the imaging measure reliably predict the effect of treatment on clinically meaningful endpoints.

### 6.1. Technical Standardization and Feasibility in Myocardial FAPI Imaging

Establishing FAPI imaging as a reproducible clinical tool requires rigorous technical harmonization. Current cardiovascular studies utilize various [^68^Ga]Ga-FAPI and [^18^F]-FAPI PET/CT—labelled compounds with distinct pharmacokinetics; because their results are not interchangeable, the specific tracer must always be identified. Similarly, quantitative metrics employed across studies —such as SUVmax, SUVmean, target-to-background ratios, and volumetric burdens—represent different biological constructs and cannot be used interchangeably. Quantification is highly sensitive to methodological variables, including uptake intervals, reconstruction algorithms, motion correction, and reference-tissue selection. Because the 2025 Society of Nuclear Medicine and Molecular Imaging/European Association of Nuclear Medicine (SNMMI/EANM) guidelines lack cardiac-specific parameters, multicenter implementation remains dependent on establishing standardized cardiovascular protocols, physiological reference ranges, and cross-scanner phantom calibrations [28]. Clinical feasibility and practical implementation of imaging platforms are limited by radiation logistics and infrastructure requirements. Cumulative ionizing radiation from PET/CT is a key concern, especially for serial imaging in younger patients with inherited cardiomyopathies. Logistically, [^68^Ga]Ga-FAPI-tracers face production-capacity limits, while [^18^F]-FAPI tracers support wider distribution but require extensive cyclotron infrastructure. Finally, while hybrid PET/CMR eliminates CT radiation and allows simultaneous tissue characterization, its widespread adoption is restricted by high costs, limited availability, and technical complexities like MR-based attenuation correction.

### 6.2. Validation of FAPI PET as an Imaging Biomarker

To establish myocardial FAPI uptake as a robust imaging biomarker, a comprehensive validation framework spanning three distinct areas is required.

Technical robustness must be established by determining intra- and interobserver reproducibility, short-term test–retest variability, and interscanner comparability. Quantitative measurements must also prove resilient to variations in image reconstruction, motion correction, segmentation, and reference-tissue selection.

Because FAPI uptake reflects active restructuring rather than total collagen volume, imaging findings cannot simply be compared to total fibrotic area. When scarce histological tissue is available (such as from myectomies or explanted hearts), FAPI signals must be validated against a multiparametric panel, including FAP immunohistochemical expression, fibroblast phenotypes, collagen maturation, inflammation, and spatially matched CMR findings. Furthermore, studies must correlate FAPI uptake with circulating biomarkers and multi-parametric CMR to define the distinct, incremental biological information PET imaging provides.

### 6.3. Other Limitations

In this review, we based our synthesis on a selection of articles that allow us to analyze data aligned with our study’s primary objective: [state objective]. Studies evaluating the application of FAPI PET/CT in patients with cardiac pathologies—particularly cardiomyopathies—remain limited, as this imaging technique is only now emerging in the field. Consequently, the literature published to date originates from a small group of investigators and remains concentrated within a limited number of institutions.

Current data are limited to small, cross-sectional cohorts and correlations with calculated risk scores, which are insufficient to prove clinical utility. Future research must utilize large, multicenter populations with prolonged follow-up to evaluate prospectively adjudicated clinical endpoints—such as SCD, ventricular arrhythmias, and cardiovascular mortality. Ultimately, FAPI PET must demonstrate incremental prognostic value beyond established variables like ventricular morphology, function or LGE burden.

## 7. Conclusions

FAP-targeted PET imaging provides a molecular perspective on myocardial remodelling by enabling the in vivo assessment of FAP-associated cellular activity. In contrast to established CMR techniques, which characterize myocardial structure, ventricular function, tissue composition, interstitial expansion, and replacement fibrosis, FAPI PET interrogates a distinct and potentially complementary component of the remodelling process. Myocardial FAPI uptake should not, however, be interpreted as a direct measure of collagen deposition, histological fibrosis, fibroblast number, or myofibroblast abundance.

Preliminary studies in HCM and other cardiovascular conditions have demonstrated spatially heterogeneous myocardial FAPI uptake, including activity in regions with and without conventional CMR abnormalities. Associations with myocardial deformation, circulating biomarkers, ventricular function, structural tissue characteristics, and calculated clinical risk support the biological relevance of the imaging signal. Nevertheless, the available evidence derives predominantly from small, exploratory, and frequently single-centre studies, with limited histopathological validation, few longitudinal cohorts, and no established prediction of adjudicated clinical outcomes.

FAPI PET therefore remains an investigational cardiovascular imaging approach. Its future clinical role will depend on tracer-specific standardization, analytical reproducibility, biological validation, definition of physiological and abnormal quantitative ranges, and demonstration of incremental diagnostic, prognostic, or pharmacodynamic value beyond established clinical assessment and CMR. Prospective multicenter studies are particularly needed to determine whether FAPI imaging can improve disease phenotyping, predict clinically meaningful outcomes, or reliably measure treatment-related changes in myocardial remodelling.

At present, the principal value of FAPI PET lies in advancing mechanistic understanding of myocardial remodelling and complementing structural, functional, and tissue-characterization imaging. Its use in routine clinical decision-making, arrhythmic risk stratification, or therapeutic monitoring will require robust prospective validation.

## Figures and Tables

**Table 1:** Comparison of CMR and FAPI PET for the assessment of myocardial remodelling. Comparative biological readouts, methodological strengths, limitations, and current clinical status of CMR and FAPI PET for the assessment of myocardial remodelling.

Evaluation Domain	CMR	FAPI PET
Principal biological results	Myocardial structure, ventricular function, focal extracellular-space expansion, diffuse tissue abnormalities, interstitial expansion, edema, and perfusion	Tissue distribution of a radiolabeled FAP inhibitor, reflecting accessible FAP expression in activated stromal cells
Principal techniques or metrics	LGE; native T1 and T2 mapping; post-contrast T1; ECV; perfusion; CMR feature-tracking strain	SUV_max, SUV_mean, target-to-background ratio, segmental uptake, uptake volume, and investigational composite activity or volume measures
Relationship with fibrosis	LGE predominantly identifies focal replacement fibrosis; native T1 and ECV provide complementary information on diffuse myocardial and interstitial abnormalities	Does not directly measure collagen or histological fibrosis; reflects FAP-associated cellular activity that may accompany active remodelling
Main strengths	High spatial resolution; accurate ventricular volumes, mass, wall thickness, and function; established myocardial tissue characterization; no ionizing radiation; extensive clinical and prognostic evidence	High molecular sensitivity; potential visualization of activated fibroblast-associated processes; may identify myocardial involvement beyond regions of hypertrophy or LGE; permits whole-body assessment
Evidence in HCM	Established role in diagnosis, phenotyping, differential diagnosis, and risk assessment; LGE extent is an acknowledged risk modifier, although thresholds and quantification methods vary	Small prospective studies show heterogeneous uptake, associations with biomarkers and calculated risk scores, and uptake in some segments without LGE or overt hypertrophy
Main biological limitations	Does not directly identify FAP-associated fibroblast activation, active collagen synthesis, or the cellular origin of matrix expansion; mapping abnormalities are not histologically specific	FAP expression is not disease-specific; uptake may reflect inflammation, ischemic repair, wound healing, fibrotic remodelling, degenerative disease, or neoplasia
Main technical limitations	Dependence on magnetic field strength, pulse sequence, reference ranges, contrast administration, segmentation, and post-processing; some patients have contraindications or reduced image quality	Lower spatial resolution; partial-volume and motion effects; dependence on tracer chemistry, uptake time, blood-pool clearance, background selection, segmentation, and reconstruction
Standardization	Relatively mature, although LGE quantification and mapping reference ranges remain method-dependent	No validated cardiac acquisition protocol, normal range, uptake threshold, reference tissue, or universally accepted quantitative endpoint
Safety and logistics	No ionizing radiation; gadolinium may be contraindicated or require caution in selected patients, although native mapping can be performed without contrast	Ionizing radiation; radiotracer production and regulatory requirements; limited availability; radiation dose varies according to tracer and CT protocol
Current clinical status	Established clinical modality	Investigational cardiovascular application
Potential complementary role	Defines anatomy, function, tissue composition, and established structural disease	May add information on active FAP-expressing cellular remodelling and support mechanistic phenotyping in research settings

Abbreviations: CMR, cardiovascular magnetic resonance; ECV, extracellular volume; FAP, fibroblast activation protein; FAPI, fibroblast activation protein inhibitor; HCM, hypertrophic cardiomyopathy; LGE, late gadolinium enhancement; PET, positron emission tomography; SUV, standardized uptake value.

**Table 2:** Principal challenges and validation requirements for cardiovascular FAPI PET.

Domain	Current Limitation	Consequence Interpretation	Required Evidence or Methodological Development
**Biological specificity**	FAP is expressed in multiple inflammatory, reparative, fibrotic, degenerative, and neoplastic environments	Uptake does not identify a unique disease or distinguish adaptive repair from pathological fibrosis	Disease-specific studies integrating clinical context, CMR, inflammatory imaging, biomarkers, and tissue analysis
**Relationship with fibrosis**	FAPI uptake does not directly quantify collagen or total ECM burden	PET signal cannot be interpreted as equivalent to histological fibrosis	Comparison with FAP immunohistochemistry, fibroblast phenotypes, collagen composition, and markers of matrix turnover
**Tracer heterogeneity**	Multiple [^68^Ga]Ga and [^18^F]F-labelled compounds with different pharmacokinetics	Results and thresholds may not be transferable between tracers	Head-to-head studies and tracer-specific reference ranges and protocols
**Quantitative metrics**	Use of SUV_max, SUV_mean, TBR, segment counts, uptake volume, and composite measures	Metrics are not interchangeable and may show different clinical associations	Prespecified standardized endpoints and validation of their biological and prognostic meaning
**Acquisition and analysis**	Variation in uptake time, reconstruction, motion correction, reference tissue, and segmentation	Reduced comparability and risk of measurement bias	Harmonized multicenter acquisition, reconstruction, quality-control, and analysis procedures
**Reproducibility**	Limited test–retest and interscanner data	Uncertainty as to whether serial changes exceed measurement variability	Test–retest studies, reproducibility analyses, and definition of minimum detectable change
**Histopathological validation**	Tissue data limited to selected surgical or advanced cases	Incomplete understanding of the cellular substrate of uptake	Prospective imaging–tissue studies using spatially matched sampling and multiparametric histology
**Prognostic evidence**	Predominantly small cohorts and associations with calculated risk scores	No established prediction of clinical events	Multicenter longitudinal studies with adjudicated arrhythmic, heart-failure, and mortality endpoints
**Therapeutic monitoring**	No controlled evidence that uptake changes represent treatment effect	Cannot currently guide treatment selection or dose adjustment	Interventional studies with untreated or comparator groups, pharmacodynamic endpoints, and clinical outcomes
**Safety and logistics**	Radiation exposure, tracer production, cost, and limited PET/CMR availability	May restrict serial imaging and broad implementation	Dose optimization, health-economic analysis, workflow assessment, and identification of clinical scenarios with clear incremental value
**Regulatory qualification**	No validated cardiac FAPI biomarker or surrogate endpoint	Not suitable for pivotal trial decisions or routine clinical algorithms	Formal analytical validation, clinical validation, and evidence linking biomarker change to patient benefit

Abbreviations: CMR, cardiac magnetic resonance; ECM, extracellular matrix; FAP, fibroblast activation protein; FAPI, fibroblast activation protein inhibitor; PET, positron emission tomography; SUV, standardized uptake value; TBR, target-to-background ratio.

**Table 3:** Selected clinical studies of FAPI-targeted imaging in hypertrophic cardiomyopathy.

Study	Design Population	Tracer and Imaging Approach	Principal Findings	Main Limitations
Wang et al., 2023 [17]	Prospective study; 50 patients with HCM and 22 healthy controls	[^18^F]F-FAPI PET/CT; CMR assessment of hypertrophy; HCM Risk-SCD score	Heterogeneous uptake extending beyond hypertrophied segments; associations with NT-proBNP, hs-cTnI, left atrial diameter, LV function, and calculated five-year SCD risk	Small and single-centre cohort; no outcomes were available because of the low occurrence rate of CVE and short follow-up period.
Zhang et al., 2023 [23]	Prospective study; 49 patients with HCM	[^18^F]F-FAPI PET/CT and CMR feature-tracking strain	More FAPI-positive segments than hypertrophied or CMR tissue-characterization-positive segments; impaired strain in some FAPI-positive/CMR-negative regions	Small sample size and single centre; segment-level analysis; low occurrence rate of CVE; no longitudinal clinical endpoints
Ding et al., 2024 [18]	Prospective integrated imaging study; 20 patients with HCM and a healthy control group	Integrated [^68^Ga]Ga-FAPI-04 PET/CMR, including LGE, mapping, ECV, and strain	FAPI uptake in 95% of patients and 156 of 320 segments; higher FAPI uptake showed a positive correlation with SCD and AF risk scores	Small and single-centre cohort; No outcomes were available
Li et al., 2024 [41]	Case series; two patients with obstructive HCM	[^18^F]F-FAPI PET/CT and Morrow myotomy procedure	Septal intense and heterogeneous uptake with a significant number of activated fibroblasts in histopathology tissue	Only two cases; selected; No outcomes were available
Song et al., 2026 [43]	Retrospective two-centre proof-of-concept study; 92 patients with HCM	Construct and internally validate a [^18^F]F-PET-derived radiomics model for predicting SCD in HCM	PET-based radiomics with FAPI showed potential for improving risk stratification in HCM	Predicted risk model from 2014 rather than clinical events; retrospective small sample; low incidence of SCD

Abbreviations: AF, atrial fibrillation; CK-MB, creatine kinase myocardial band; CMR, cardiovascular magnetic resonance; CVE, cardiovascular events; ECV, extracellular volume; FAPI, fibroblast activation protein inhibitor; HCM, hypertrophic cardiomyopathy; hs-cTnI, high-sensitivity cardiac troponin I; LGE, late gadolinium enhancement; LV, left ventricular; NT-proBNP, N-terminal pro-B-type natriuretic peptide; PET, positron emission tomography; SCD, sudden cardiac death.

## Data Availability

No new data were created or analyzed in this study.
